# Comparison of the genomic alterations present in tumor samples from patients with metastatic inflammatory *versus* non-inflammatory breast cancer reveals AURKA as a potential treatment target

**DOI:** 10.1016/j.breast.2023.01.010

**Published:** 2023-01-25

**Authors:** François Richard, Maxim De Schepper, Marion Maetens, Sophia Leduc, Edoardo Isnaldi, Tatjana Geukens, Karen Van Baelen, Ha-Linh Nguyen, Peter Vermeulen, Steven Van Laere, François Bertucci, Naoto Ueno, Luc Dirix, Giuseppe Floris, Elia Biganzoli, Christine Desmedt

**Affiliations:** a Laboratory for Translational Breast Cancer Research, Department of Oncology, KU Leuven, 3000, Leuven, Belgium; b Department of Internal Medicine and Medical Specialties, University of Genoa, IT-16132, Genoa, Italy; c Translational Cancer Research Unit, GZA Hospitals & CORE, MIPRO, University of Antwerp, Antwerp, Belgium; d Department of Oncological Research, Oncology Center, GZA Hospitals Sint-Augustinus, Antwerp, Belgium; e Center for Oncological Research (CORE), Integrated Personalized and Precision Oncology Network (IPPON), University of Antwerp, Belgium; f Institut Paoli Calmettes, CRCM, INSERM U1068, CNRS UMR7258, Aix-Marseille Université, Marseille, France; g Department of Breast Medical Oncology, Division of Cancer Medicine, The University of Texas MD Anderson Cancer Center, Houston, TX, USA; h Department of Imaging and Pathology, Laboratory of Translational Cell & Tissue Research and University Hospitals Leuven, KU Leuven, 3000, Leuven, Belgium; i Unit of Medical Statistics, Biometry and Epidemiology, Department of Biomedical and Clinical Sciences (DIBIC) & DSRC, Ospedale “L. Sacco” LITA Campus, Università degli Studi di Milano, 20157, Milan, Italy

**Keywords:** Inflammatory breast cancer, Metastatic breast cancer, Genomic alterations, AURKA-Inhibitors

## Abstract

Inflammatory breast cancer (IBC) is a rare but aggressive subtype of breast cancer, mainly characterized using primary tumor samples. Here, using public datasets, we compared the genomic alterations in primary and metastatic samples from patients with metastatic IBC versus patients with metastatic non-IBC. We observed a higher frequency of *AURKA* amplification in IBC. We further showed that *AURKA* amplification was associated with increased *AURKA* mRNA expression, which we demonstrated was higher in IBC. Finally, higher protein expression of AURKA was associated with worse prognosis in patients with IBC. These findings deserve further investigation given the existence of AURKA-inhibitors.

## Highlights

• Inflammatory breast cancer (IBC) is a rare understudied but aggressive type of breast cancer.
• *AURKA* amplification appeared to be more common in patients with metastatic IBC as compared to non-IBC.
• *AURKA* amplification is associated with AURKA expression.
• AURKA protein expression is associated with worse prognosis in patients with IBC.

## 1 Introduction

Inflammatory breast cancer (IBC) represents a rare (∼3% of all invasive breast cancers) but very aggressive form of breast cancer [1]. Patients with IBC are generally younger, have more frequent axillary lymph node involvement and are more frequently diagnosed with upfront stage IV disease as compared to non-IBC patients [2]. With regard to the distribution of the molecular subtypes, 75% of the IBC tumors are classified in the more aggressive basal-like, HER2-positive and luminal B subtypes, as opposed to only 54% for non-IBC [3]. Patients with IBC generally have a worse survival than patients with non-IBC [2], potentially restricted to the triple-negative subtype [4]. Despite improvement of treatment regimens for breast cancer in time, patients with IBC are often excluded from trials investigating targeted therapies, and specific therapies for IBC are lacking. During the last two decades, researchers have attempted to provide a description of these tumors at the molecular level (as reviewed by Costa et al. [5]). Often the genomic landscape of IBC was characterized using targeted or whole-exome sequencing [[6], [7], [8], [9], [10], [11], [12], [13]]. However, none of them used patient-matched normal DNA for somatic mutation calling and the comparative analyses with non-IBC tumors generally did not take into consideration the differences in stage and molecular subtypes observed between IBC and non-IBC patients, with the exception of one study [12] having both. Here we aimed at characterizing the genomic alterations present in metastatic IBC using publicly available data and validating one of our main findings on samples from an institutional series of patients with IBC.

### 1.1 Patient and methods

**Fig. 1:**
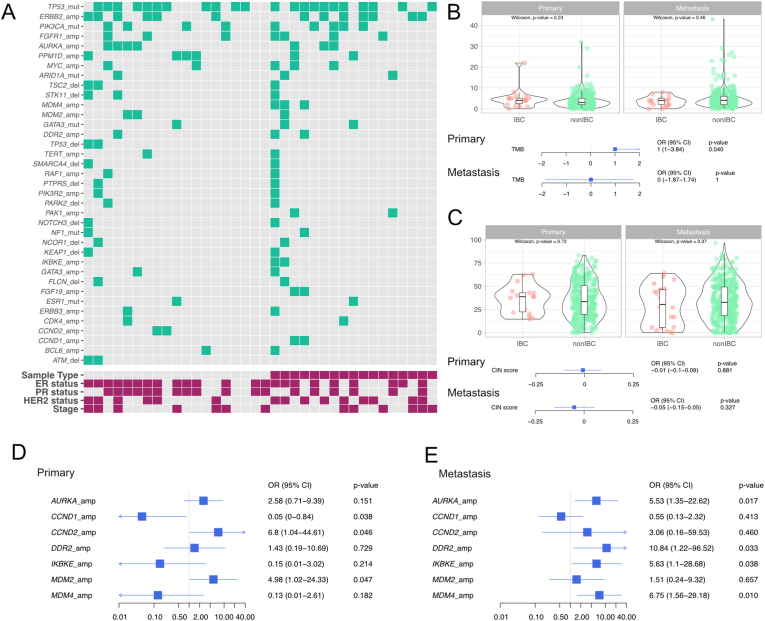
Genomic alteration landscape in patients with recurrent IBC compared to patients with recurrent non-IBC. **(A)** Heatmap of the IBC cohort. Only alterations occurring at least twice in patients with IBC are shown and ordered according to their prevalence. A patient was considered as ‘having’ the alteration if at least one of her samples were altered (4 patients with IBC had multiple samples in the metastatic setting). Patients are grouped per sample type, with primaries on the left and metastases on the right. Green and grey depicts presence and absence of the alteration respectively. The lower panel refers to the clinicopathological data with Sample Type (grey:Primary; red:Metastasis), ER status (Negative; Positive), PR status (Negative; Positive), HER2 status (Negative; Positive), Stage (III; IV). **(B,C)** Violins and forest plots representing the association between BC type (IBC *vs* non-IBC) and the TMB (B) or CIN score (C) as linear regression and quantile regression respectively adjusted for HR status (positive *vs* negative), HER2 status (positive *vs* negative), stage (IV *vs* I, III *vs* I, II *vs* I) and cohort (MBC *vs* MSK). The median is taken in case of multiple samples per patient. **(D, E)** Forest plot representing the association between BC type (IBC *vs* non-IBC) and the genomic alteration in primaries (D) and metastases (E) as firth logistic regression adjusted for HR status (positive *vs* negative), HER2 status (positive *vs* negative), stage (IV *vs* I, III *vs* I, II *vs* I) and cohort (MBC *vs* MSK). Alterations significantly associated at least in one of the panels are shown in both. (A, D, E) Only “Oncogenic”, “Likely Oncogenic”, and “Predicted Oncogenic” alterations are shown. Abbreviations: amp: amplification, CI: confidence interval, CIN: chromosomal instability, del: deletion, ER: estrogen receptor, HR: hormonal receptor, IBC: inflammatory breast cancer, mut: mutation, NST: non special type, OR: odds ratio, PR: progesterone receptor, TMB: tumor mutational burden. . (For interpretation of the references to colour in this figure legend, the reader is referred to the Web version of this article.)

We considered female patients with invasive breast cancer of no special type (NST, commonly referred to as invasive ductal breast cancer) who were either upfront metastatic or relapsed later after primary diagnosis from the Memorial Sloan Kettering-IMPACT [14] and the Metastatic Breast Cancer project (MBC) [15] cohorts.: 34 patients with IBC (including 2 with both primary and metastatic samples, Fig. 1A) and 602 with non-IBC (including 33 with both primary and metastatic samples) (Supplementary Table 1). We further investigated AURKA expression at the mRNA and protein level in the dataset of Van Laere et al. [3] (n = 67 IBC and 169 non-IBC) and in an institutional cohort of patients with IBC (n = 43 patients), respectively. We applied Firth logistic regression adjusted for hormonal receptor status (positive *vs* negative), HER2 status (positive *vs* negative), stage (II *vs* I, III *vs* I, IV *vs* I) and cohort (MSK-IMPACT *vs* MBC) with genomic alterations or metastatic sites as outcome variables and IBC status as independent variable (yes *vs* no). Methods are further detailed in the Supplementary Appendix.

## 2 Results

Metastatic samples from chest wall and skin metastases were significantly more represented in patients with IBC as compared to patients with non-IBC (Adjusted Odds Ratio (OR_adj_): 6.04, 95% confidence interval (95CI): [1.91–17.94], p-value: 0.003 and OR_adj_: 7.43, 95CI: [1.63–29.30], p-value: 0.012, respectively Supplementary Table 2), which is in line with the course of the IBC disease [16].

Several studies have suggested that tumors from IBC patients have a higher tumor mutational burden (TMB) than tumors from non-IBC patients [9,10]. Here we confirmed an increase in TMB in primary tumors from IBC *versus* non-IBC patients (Adjusted coefficient (coef_adj_): 1.00, 95CI: [1.00–3.84], p-value: 0.040), but not in metastases (coef_adj_ metastases: 1.11, 95CI: [−1.87-1.74], p-value: 1) (Fig. 1B, and Supplementary Table 3). However, we did not observe a difference in chromosomal instability (CIN) score in patients with IBC as compared to patients with non-IBC (Fig. 1C, and Supplementary Table 4).

**Fig. 2:**
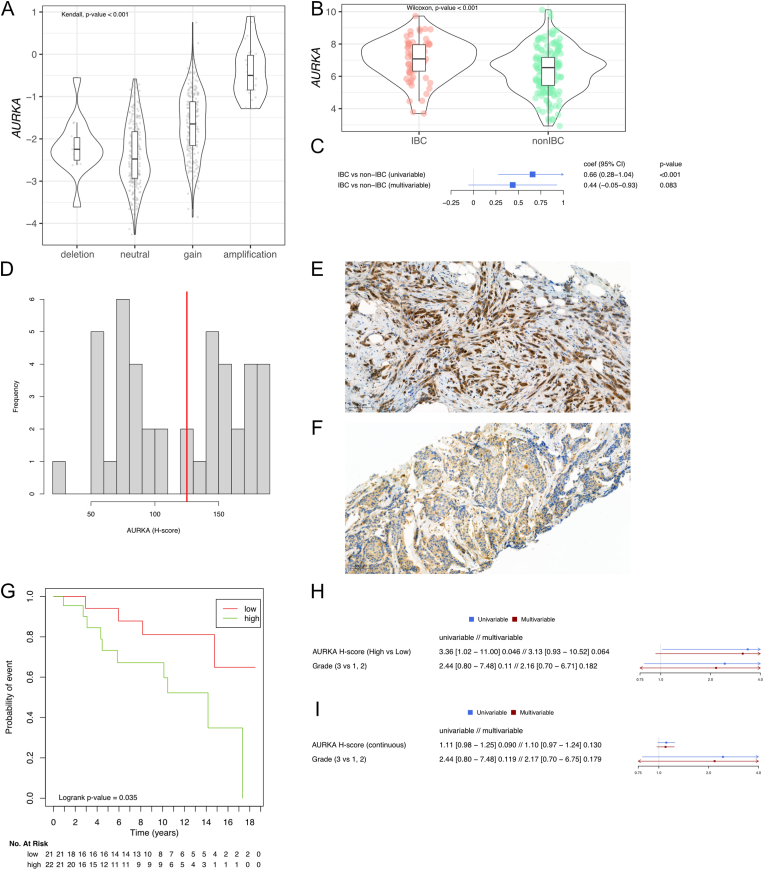
**(A)** Normalized *AURKA* expression according to *AURKA* copy number events in the patients with NST, ER-positive/HER2-negative BC in the TCGA dataset. **(B,C)** Normalized *AURKA* expression according to BC type in the patients with ER-positive/HER2-negative BC in the E-MTAB-1006 dataset. **(D)** H-score distribution in the institutional cohort. The median of 125 is depicted in red. **(E,F)** Immunohistochemical AURKA staining for 2 cases of the institutional cohort. H-scores are of 176 and 88 for E and F respectively. **(G)** Kaplan-Meier curves for OS in the institutional cohort, regarding AURKA expression categorized around its median. **(H, I)** Forest plots for the cox regressions regarding OS in univariable and multivariable models, considering AURKA by category (high vs low around the median) (H) or continuous with a 10-unit increase (I). For each variable, hazard ratio is reported, followed by the 95% CI, and the p-value. (G, H, I) 14 events were observed in the analysis for OS. (C, H, I) P-values are from Wald tests. Abbreviations: BC: breast cancer, CI: confidence interval, coef: coefficient, ER: estrogen receptor, IBC: inflammatory breast cancer, NST: non special type, OS: overall survival. . (For interpretation of the references to colour in this figure legend, the reader is referred to the Web version of this article.)

We further focused on the potentially clinically and biologically relevant genomic alterations present in IBC according to OncoKB [17] (Fig. 1A). In IBC, the top mutated genes in the samples from the primary tumor samples were *TP53* (11/19, 58%) and *PIK3CA* (4/19, 21%), and in the metastatic samples *TP53* (11/17, 65%), *PIK3CA* (5/17, 29%), then *ARID1A* and *GATA3* (2/17, 12%). While the mutational frequencies of *TP53* and *PIK3CA* are in line with previous reports [[6], [7], [8], [9], [10], [11], [12]], we did not detect *ERBB2* or *ERBB3* mutations as in Matsuda et al. [9] or Hamm et al. [7], respectively. Concerning copy number aberrations (CNAs), the top amplified genes in the samples from the primary tumor were *ERBB2* (6/19, 32%), *AURKA, PPM1D* (4/19, 21%), and *FGFR1* (3/19, 16%), while in the metastatic samples *ERBB2* (7/17, 41%), *CDK12, FGFR1* (4/17, 24%), and *AURKA, MDM4, MYC* (3/17, 18%). We further compared the prevalence of mutations and CNAs in IBC to non-IBC using regression analyses adjusted for stage at diagnosis and molecular subtype. We observed a numerical increase of *TP53* mutations in IBC as compared to non-IBC samples, as suggested by previous reports [[9], [10], [11], [12]], the difference was however not significant anymore in our adjusted analysis. In terms of CNAs, our analyses revealed a consistent trend in primary and metastatic samples of increased frequency of *AURKA* amplifications in patients with IBC *versus* patients with non-IBC (Fig. 1D and E, Supplementary Table 5 and Supplementary Table 6). *CCND2* and *MDM2* amplifications were also significantly more frequent in patients with IBC compared to patients with non-IBC in the primary samples, while *DDR2*, *IKBKE* and *MDM4* amplifications showed a significant association in the metastases. Of note, in our series, 5 out of the 7 *AURKA*-amplified tumors were estrogen receptor (ER)-positive and all but one harbored *TP53* mutations (Fig. 1A). Since transcriptomic data were only available for few patients from the studied cohorts, we investigated the correlation between copy number and gene expression levels using publicly available data from The Cancer Genome Atlas [18]. We observed that breast tumors with *AURKA* amplification had significantly higher gene expression levels than tumors without amplification (p-value<0.001, Fig. 2A). We then explored the expression levels of AURKA in the dataset from Van Laere et al. 2013 [3], confirming the increase of *AURKA* mRNA expression within patients with IBC as compared to patients with non-IBC, in line with previous reports from patients [19] and cell lines data [20] (Fig. 2 B,C and Supplementary Table 7). Finally, we evaluated the expression of AURKA at the protein level using immunohistochemistry (IHC) in our retrospective institutional cohort of 43 patients with ER-positive IBC. AURKA displayed a wide range of expression in this cohort (median H-score: 125, interquartile range: 75, Fig. 2 D, E, F) and higher AURKA expression was associated with worse overall survival at univariable level when considering AURKA expression by category around the median (Hazard Ratio: 3.36, 95CI: [1.02–11], p-value: 0.046 Fig. 2 G, H). The direction of the association was conserved but not significant after adjusting for grade (Fig. 2 H) or when considering AURKA expression as a continuous variable (Fig. 2 I).

## 3 Discussion

In our study we have investigated the genomic landscape of patients with either upfront metastatic or relapsed IBC. The novelty comes from: (i) the availability of germline DNA for ensuring adequate somatic calling of the mutations, (ii) the adjustment for stage and molecular subtype for the comparison between IBC and non-IBC samples, and (iii) the fact that primary and metastatic samples were studied separately. Two main findings emerged. First, in line with previous studies, we reported a higher mutational burden in IBC *versus* non-IBC samples, although only in the primary tumor samples. Second, we observed a higher prevalence of *AURKA* amplification in IBC *versus* non-IBC samples. Of note, *AURKA* was not included, or available for few samples, in the targeted sequencing panel of many studies [[9], [10], [11], [12]], potentially explaining why this observation was not made earlier. We have further shown that *AURKA* amplification is correlated with *AURKA* overexpression, and *AURKA* mRNA overexpression has previously been shown to be associated with tumor proliferation, tumorigenesis, clinical aggressiveness, and tumor progression [21] in different cancer types including breast cancer [22,23]. However, Staff et al. [24] showed that AURKA expression as assessed by IHC was not strongly associated with the Ki67 proliferation marker (Spearman's rho = 0.39). In our study, we further investigated AURKA expression by IHC in a series of samples from primary IBC and observed that higher expression levels were associated with poor prognosis, underlying a possible more complex role of AURKA on tumor progression. Our present observation could be of potential clinical relevance given the existence of several AURKA inhibitors that are currently being tested in patients with breast cancer (NCT02187991, phase 2 with alisertib, and NCT02134067, phase 1 with TAS-119). These inhibitors have already shown promising results, especially in patients with ER-positive breast cancer [22,25,26]. In our dataset, 5 out of 7 *AURKA-*amplified IBC were ER-positive, suggesting that AURKA inhibition could potentially be explored in priority as therapeutic option for patients with ER-positive IBC. The clinical relevance of the higher TMB observed in primary IBC compared to primary non-IBC should be further investigated in patients with IBC treated with immune checkpoint inhibitors, in the PELICAN trial for instance (NCT03515798).

The main limitations of our study are related to its retrospective nature, precluding centralized revision of IBC diagnosis, and to the relatively small number of IBC samples due to its rare nature, requesting future validation of the findings. Nevertheless, the IBC samples reported here were originating from two of the largest initiatives collecting and genomically characterizing samples from patients with metastatic breast cancer, emphasizing the difficulty in assembling larger series from rarer disease entities such as IBC. There is therefore a need to further collect and characterize IBC samples, something that will be achieved by increasing patient participation from the IBC community to important initiatives such as the Metastatic Breast Cancer project (www.mbcproject.org) [15,27] or post-mortem tissue donation programs, such as the one we recently developed in our institution (NCT04531696).

## 4 Availability of data and materials

Public data used for this study are available as supplementary material in Razavi et al., 2018 [14], on cBioPortal (https://www.cbioportal.org/study/summary?id=brca_mbcproject_wagle_2017) and under the accession number E-MTAB-1006 on the ArrayExpress data portal (09/2021). The public datasets along with the R code generated and analyzed during the current study are available in the CodeOcean repository, doi: https://doi.org/10.24433/CO.6792314.v1. The institutional cohort and the code related to its analysis are available upon request after author approval.

## Funding

The PhD grant from TG and the post-doc mandate from FR are supported by The Research Foundation – Flanders (10.13039/501100003130FWO). Research on IBC in the lab from 10.13039/100011639CD is supported by a grant from 10.13039/501100003130FWO (G059821 N) and from the Luxemburg Cancer Foundation (FC/2018/07). KVB and MSD are supported by the Funds Nadine de Beauffort. The post-doc mandate from GF is supported by the 10.13039/100012324University Hospitals of Leuven (KOOR 2021).

## Authors’ contributions

CD and FR designed the study. CD, FB, LD, NU, SVL provide study materials or patients’ data. EB and FR performed the statistical analyses. CD, EB, EI, FR, GF, HLN, KVL, MDS, MM, PV, SL, TG analyzed and interpreted the data. CD, EB and FR wrote the manuscript. All authors read and approved the final manuscript.

## Declaration of competing interest

The authors declare that they have no competing interests.
