# Supplementary material for: Comparison of the genomic alterations present in tumor samples from patients with metastatic inflammatory versus non-inflammatory breast cancer reveals AURKA as a potential treatment target
Source: Breast. 2023 Jan 25;69:476–80. doi: 10.1016/j.breast.2023.01.010 (PMC10300569; doi:10.1016/j.breast.2023.01.010)
Supplement: Multimedia component 2 [file mmc2.docx]

Supplementary Appendix

**Comparison of the genomic alterations present in tumor samples from patients with metastatic inflammatory *versus* non-inflammatory breast cancer reveals AURKA as a potential treatment target**

François Richard, Maxim De Schepper, Marion Maetens, Sophia Leduc, Edoardo Isnaldi, Tatjana Geukens, Karen Van Baelen, Ha-Linh Nguyen, Peter Vermeulen, Steven Van Laere, François Bertucci, Naoto Ueno, Luc Dirix, Giuseppe Floris, Elia Biganzoli, Christine Desmedt

Table of Contents

[Supplementary Methods 2](#_Toc120805051)

[Patient selection – Publicly available datasets 2](#_Toc120805052)

[Patient selection – Institutional cohort 2](#_Toc120805053)

[Immunohistochemistry (IHC) 2](#_Toc120805054)

[Evaluation of immunohistochemical staining 3](#_Toc120805055)

[Statistical analyses 3](#_Toc120805056)

[References 4](#_Toc120805057)

# Supplementary Methods

## Patient selection – Publicly available datasets

We retrieved the publicly available clinical and genomic data from primary and metastatic samples from MSK-IMPACT [1] and the MBC project[2] from cbioportal (09/21). Inflammatory Breast Cancer (IBC) was defined as cT4d and “PRD Ever Inflammatory”=YES respectively. Given the rarity of lobular histology in IBC[3], we considered only non special type (NST) carcinomas in both cohorts. Of note, a germline matched DNA was available for the calling of somatic mutations in both cohorts. The TCGA dataset was downloaded from cbioportal (09/21) while the cases classified as estrogen receptor (ER)-positve/HER2-negative High Prolif and ER-positive/HER2-negative Low Prolif were retrieved from Van Laere, *et al.* 2013[4] and denominated as the E-MTAB-1006 dataset.

## Patient selection – Institutional cohort

A series of 43 patients with ER-positive/HER2-negative IBC were retrospectively collected from the biobank of the University Hospitals Leuven after ethical committee approval (S-62499). Diagnosis dates were ranging from 02/2002 to 09/2008, median age was 53.40 years (Interquartile range: 12.18 range: [28-71]). There were 2, 24 and 17 patients with tumor of grades 1, 2, 3 respectively. We focused on ER-positive IBC because we observed that *AURKA* amplifications were mainly present in patients with ER-positive IBC (5/7 patients with *AURKA* amplification were having ER-positive tumors).

## Immunohistochemistry (IHC)

Formalin fixed paraffin embedded blocks from diagnostic core needle biopsies of the 43 ER-patients positive/HER2-negative IBC were retrieved from the University Hospitals Leuven biobank. 3,5µm thick sections were immunohistochemically stained for Anti-Aurora A antibody (EP1008Y, ab52973, Abcam, Cambridge, UK) with dilution 1:100 on Leica BOND MAX. Antigen retrieval was performed with citrate buffer, pH6.

## Evaluation of immunohistochemical staining

AURKA was scored as a continuous variable using the H-score (score 0: no staining; score 1: weak staining; score 2: moderate staining, score 3: strong staining). Positive staining was defined as cytoplasmic and/or nuclear staining.

## Statistical analyses

Alterations were annotated using oncotator [5] to identify potential driver events (annotated as "Predicted Oncogenic", "Likely Oncogenic", or "Oncogenic"). Unless otherwise specified, the set of alterations was restricted to these driver events. A minimum of 5 patients harboring the alterations were required to report the analysis. In case of an odds ratio (OR) greater than 1, at least 2 events in the IBC group were requested for the association to be reported. Chromosomal instability (CIN) score and tumor mutation burden (TMB) were investigated by linear regression and quantile regression respectively, adjusted for hormonal receptor status (positive *vs* negative), HER2 status (positive *vs* negative), stage (II *vs* I, III *vs* I, IV *vs* I) and cohort (MSK-IMPACT *vs* MBC). The median was considered in the quantile regression. CIN score was extracted from the public data, while TMB was computed as the total number of driver and passenger mutations. Primary and metastatic samples were analyzed separately, a patient was considered as ‘having’ the alteration if at least one of her samples were altered. In 5 patients the stage was inferred to III in the MBC cohort given the curative intent of the lumpectomy/mastectomy they received. In 2 patients, the HER2 status was inferred from the copy number status, therefore the HER2 status was not retained in the copy number association analysis. P-values were 2-sided. In the E-MTAB-1006 dataset, Firth logistic regressions were adjusted for stage (II *vs* I, III *vs* I, IV *vs* I) in the multivariable model. All analyses were performed in R 4.2.1.

# References

[1] Razavi P, Chang MT, Xu G, Bandlamudi C, Ross DS, Vasan N, et al. The Genomic Landscape of Endocrine-Resistant Advanced Breast Cancers. Cancer Cell 2018;34:427-438.e6. https://doi.org/10.1016/j.ccell.2018.08.008.

[2] Wagle N, Painter C, Anastasio E, Dunphy M, McGillicuddy M, Kim D, et al. The Metastatic Breast Cancer (MBC) project: Accelerating translational research through direct patient engagement. J Clin Oncol 2017;35:1076–1076. https://doi.org/10.1200/jco.2017.35.15_suppl.1076.

[3] Raghav K, French JT, Ueno NT, Lei X, Krishnamurthy S, Reuben JM, et al. Inflammatory breast cancer: A distinct clinicopathological entity transcending histological distinction. PLoS One 2016;11:e0145534. https://doi.org/10.1371/journal.pone.0145534.

[4] Van Laere SJ, Ueno NT, Finetti P, Vermeulen P, Lucci A, Robertson FM, et al. Uncovering the molecular secrets of inflammatory breast cancer biology: An integrated analysis of three distinct affymetrix gene expression datasets. Clin Cancer Res 2013;19:4685–96. https://doi.org/10.1158/1078-0432.CCR-12-2549.

[5] Chakravarty D, Gao J, Phillips S, Kundra R, Zhang H, Wang J, et al. OncoKB: A Precision Oncology Knowledge Base. JCO Precis Oncol 2017;1:1–16. https://doi.org/10.1200/po.17.00011.
